# Adverse events of SARS-CoV-2 vaccination in patients with inflammatory rheumatic disease during repeated vaccination: An observational cohort study

**DOI:** 10.1007/s00296-025-06013-z

**Published:** 2025-10-23

**Authors:** Liam Huppke, Christina Gebhardt, Lea Grümme, Julia Lichtnekert, Delila Singh, Fabian T. H. Ullrich, Stefan Wolfrum, Alla Skapenko, Hendrik Schulze-Koops

**Affiliations:** Division of Rheumatology and Clinical Immunology, Department of Medicine IV, LMU University Hospital, 80336 Munich, Germany

**Keywords:** SARS-CoV-2, COVID-19 Vaccines, Control groups, Rheumatology, Autoimmune diseases, Risk factors, Immunosuppression therapy, Inflammation

## Abstract

To give further insights into the safety of SARS-CoV-2 vaccinations in patients with inflammatory rheumatic diseases (IRDs) compared to healthy individuals and to highlight changes over the course of repeated vaccinations. In this single-centre study, SARS-CoV-2 vaccinated IRD patients were recruited from the hospital of the University of Munich. Healthcare workers served as the control group. Adverse events following each vaccination were assessed using questionnaires. Descriptive statistics and non-parametric tests were used to illustrate the differences between IRD patients and the control group. Between January 1, 2021, and Septemper 30, 2022, 235 IRD patients (60.4% female) and 102 healthy individuals (66.7% female) were enrolled in this study. The frequency of patients who experienced adverse events after the first vaccination (140 [59.6%]) was significantly lower compared to the control group (86 [84.3%]) (OR = 0.274 [95% CI: 0.151–0.497]; *P* < 0.0001). The same was true after the second vaccination (patients: 138 [58.7%]; controls: 80 [78.4%]) (OR = 0.391 [0.228–0.670]; *P* < 0.001), and after the third vaccination (123 [56.4%]; 70 [69.3%]) (OR = 0.573 [0.348–0.946]; *P* = 0.029). Local side effects occurred with similar frequency in both groups. However, systemic effects occurred significantly less frequently in patients after all vaccinations than in controls (1. vaccination 97 [41.3%] to 61 [59.8%]: OR = 0.472 [0.294–0.759], *P* = 0.002; 2. vaccination 91 [38.7%] to 60 [58.8%]: OR = 0.442 [0.275–0.710], *P* < 0.001; 3. vaccination: 85 [39.0%] to 52 [51.5%]: OR = 0.602 [0.374–0.969], *P* = 0.036). SARS-CoV-2 vaccines were well tolerated by patients with IRDs. They experienced systemic side effects less frequently in patients than in healthy controls, suggesting a possible association between IRDs/immunosuppressive therapies and attenuation of vaccination reactions. Further research is needed to determine the cause of these differences.

**Supplementary Information:**

The online version contains supplementary material available at 10.1007/s00296-025-06013-z.

## Introduction

Active inflammatory rheumatic diseases (IRDs) and some of the therapies used to treat such autoimmune diseases are just two examples of the many potential risk factors that have been linked to a severe course of a severe acute respiratory syndrome coronavirus-2 (SARS-CoV-2) infection [1, 2]. Finding a way to protect the individuals at risk, as well as healthy people, from infection and severe disease was of paramount importance. In late 2020, the first vaccines against SARS-CoV-2 were approved. This involved the use of a new principle: vaccines based on mRNA. While this new vaccine technology was an important reason for the rapid development of vaccines against SARS-CoV-2, there were, by their nature, no studies evaluating the safety of the vaccines in at-risk groups. In the general population, adverse reactions to vaccination were similar to other vaccines, with pain at the injection site and fatigue being the most common adverse events [3]. Serious adverse events occurred infrequently. However, data on potential interactions of the vaccines with IRDs or with their therapies were not available because patients with IRDs were excluded from the pivotal trials [3–5]. An extensive study with over 10,000 participants from all over the world has shown that the vaccination is in fact safe for patients with IRDs and the risk of adverse events is not much different to healthy individuals [6]. With such a large heterogenic study population the prevalence of severe or seldomly occurring adverse events as well as the general safety of the vaccines can be depicted very well. However, due to the heterogeneity of an international study population, such a study is less likely to collect some effects that do occur but are perceived, assessed, and reported differently in different societies with different medical care, thereby potentially diluting the signal strength and masking the differences of these events.

Given that on the one hand the global pandemic is not yet over, with some severe and even fatal cases of SARS-CoV-2 infection in particular in patients with IRDs undergoing immunosuppressive therapy and on the other hand the worrying observation of a noticeable reluctance among the general population and our patients to receive another protective SARS-CoV-2 vaccination, the delineation of the risks of vaccination for patients in direct comparison to healthy individuals is necessary and useful for the patients and their treating physicians. We therefore present data on the safety and tolerability of the first three SARS-CoV-2 vaccinations administered since the start of the global vaccination campaign, comparing patients with IRD and a healthy control cohort from the LMU Munich University Hospital. The data show that vaccination is safe in patients with IRDs and that patients actually experience fewer systemic side effects from vaccination compared to healthy control subjects. The results are reassuring and support patients and their treating physicians in improving care.

## Patients and methods

### Study design and population

In this single-centre study, questionnaires were used to assess adverse events occurring after vaccination in patients with IRDs.

Between Jan 1, 2021, and Sep 30, 2022, patients with IRDs who were vaccinated against SARS-CoV-2 were recruited from the outpatient clinic of the Division of Rheumatology and Clinical Immunology at the hospital of the LMU Munich. Health care workers were recruited as healthy controls. In this later group, autoimmune diseases or immunosuppressive therapies were defined as exclusion criteria.

All participants had to fill out a questionnaire regarding age, dates of vaccination against SARS-CoV-2, and vaccines used. Next was a list of the most common adverse events (Table 1) occurring after vaccination, where they had to mark the adverse events they experienced after each vaccination and note the time after vaccination when they occurred. The option to add adverse events not presented in the list was granted. Patients were also asked to provide information about their IRD and immunosuppressive therapy as well as to report any activation of the IRD after vaccination. The study was approved by the ethics committee of the LMU Munich (18 march, 2021; protocol number 21–0118) and all participants gave written informed consent.

**Table 1:** Adverse events after SARS-CoV-2 vaccination in patients and healthy controls

	patients			healthy controls		
adverse events	1. Vaccination	2. Vaccination	3. Vaccination	1. Vaccination	2. Vaccination	3. Vaccination
	*n* = 235	*n* = 233	*n* = 218	*n* = 102	*n* = 102	*n* = 101
local reactions, % (n)						
any	49.8 (117)	48.9 (115)	47.7 (104)	66.7 (68)	57.8 (59)	49.5 (50)
pain at the injection site	46.4 (109)	47.2 (110)	45.0 (98)	65.7 (67)	55.9 (57)	47.5 (48)
swelling at the injection site	12.3 (29)	9.9 (23)	13.3 (29)	10.8 (11)	10.8 (11)	9.9 (10)
redness at the injection site	8.9 (21)	7.3 (17)	8.7 (19)	7.8 (8)	7.8 (8)	6.9 (7)
systemic reactions, % (n)						
any	41.2 (97)	38.7 (91)	39.0 (85)	59.8 (61)	58.8 (60)	51.5 (52)
fatigue	29.4 (69)	27 (63)	28.0 (61)	42.2 (43)	45.1 (46)	37.6 (38)
chills	6.8 (16)	8.2 (19)	6.4 (14)	12.7 (13)	15.7 (16)	5.9 (6)
fever	5.5 (13)	6.4 (15)	6.0 (13)	12.7 (13)	16.7 (17)	8.9 (9)
headache	15.3 (36)	12.9 (30)	13.3 (29)	22.5 (23)	19.6 (20)	21.8 (22)
nausea	2.6 (6)	2.1 (5)	3.7 (8)	2.9 (3)	3.9 (4)	0 (0)
vomiting	0.4 (1)	0 (0)	0.5 (1)	0 (0)	2.0 (2)	0 (0)
diarrhea	0(0)	1.7 (4)	0.9 (2)	1.0 (1)	1.0 (1)	1.0 (1)
muscle pain	13.2 (31)	12.0 (28)	13.8 (30)	26.5 (27)	25.5 (26)	22.8 (23)
joint pain	6.8 (16)	5.2 (12)	6.4 (14)	10.8 (11)	11.8 (12)	8.9 (9)
itching	2.6 (6)	2.6 (6)	2.8 (6)	0 (0)	0 (0)	0 (0)
allergic reaction	0 (0)	0.4 (1)	0.5 (1)	0 (0)	1.0 (1)	1.0 (1)
others*						
changes in the menstrual cycle	-	-	-	-	-	1.0 (1)
subjective heart raising	0.8 (2)	0.4 (1)	0.5 (1)	-	-	-
activation of the IRD	1.3 (3)	-	0.5 (1)	-	-	-
breathing problems	-	-	0.5 (1)	-	-	-

*adverse events added by study participants

### Statistical analysis

Adverse events after vaccination were divided into local, i.e. occurring at the injection site, and systemic, i.e. occurring independently of the local reaction. For further analysis, the patients were divided into subgroups according to the underlying IRD or medication.

Categorical data were described in absolute and relative frequencies when appropriate. Descriptive statistics such as the Pearson-Chi^2^-test and odds ratio (OR) with 95% confidence interval (95% CI) were used to assess differences between patients and healthy controls. Non-parametric tests such as the Wilcoxon-test were used to show changes in groups as a function of vaccination. P-values < 0.05 were considered statistically significant. All analysis were performed with SPSS Statistics 28, and graphs were created with GraphPad Prism 9.

## Results

238 patients with various IRDs filled out the questionnaire for adverse events experienced after the SARS-CoV-2 vaccinations. Three of those had to later be excluded because vaccination against SARS-CoV-2 had not taken place. The mean age of the 235 patients left was 54 (± 15) years and 60.4% (142) were female. Most common IRDs were subtypes of arthritis (61.3%) followed by vasculitis (15.7%) (Table 2).

**Table 2:** Demographic characteristics of the study population and vaccine information

	patients	healthy controls
n	235	102
age (mean ± SD)	54 ± 15	48 ± 16
female, % (n)	60.4 (142)	66.7 (68)
IRD, % (n):		
rheumatoid arthritis	22.6 (53)	-
psoriatic arthritis	15.7 (37)	-
spondyloarthropathy	8.1 (19)	-
juvenile idiopathic arthritis	2.6 (6)	-
polymyalgia rheumatica	2.6 (6)	-
undifferentiated polyarthritis	2.1 (5)	-
SAPHO syndrome	1.3 (3)	-
other arthritides	6.4 (15)	-
systemic lupus erythematosus	7.2 (17)	-
sjögren’s syndrome	3.9 (9)	-
systemic sclerosis	0.9 (2)	-
myositis	0.9 (2)	-
other soft connective tissue disorders	1.3 (3)	-
granulomatosis with polyangiitis	7.2 (17)	-
hypereosinophilic syndrome	0.9 (2)	-
giant cell arteritis	2.6 (6)	-
takayasu arteritis	2.6 (6)	-
other vasculitides	2.6 (6)	-
other IRDs	8.9 (21)	-
Immunomodulatory therapy, % (n)		
methotrexate	28.5 (67)	-
rituximab	9.8 (23)	-
TNF inhibitors	24.7 (58)	-
IL-6R inihibitors	6.8 (16)	-
JAK- nhibitors	3.8 (9)	-
hydroxychloroquine	9.4 (22)	-
glucocorticoids	20.4 (48)	-
Vaccine, %		
1. *Vaccination*		
BNT162b2	79.0	82.4
mRNA-1273	8.5	4.9
ChAdOx1	10.3	11.8
JNJ-78,436,735	2.2	1.0
2. *Vaccination*		
BNT162b2	84.5	90.3
mRNA-1273	11.4	6.9
ChAdOx1	4.1	2.9
3. *Vaccination*		
BNT162b2	69.5	85.1
mRNA-1273	21.8	13.9
no third vaccination	7.7	1.0

The healthy control group (HCG) consisted of 102 health care workers. Mean age was 48 (± 16) years and 66.7% (68) were female.

The most frequently used vaccine in both groups was BNT162b2 (rINN tozinameran, Pfizer/BioNTech)) (70–85% in patients, 82–90% in controls). mRNA-1273 (elasomeran, Moderna) was used nearly twice as often in patients compared to the controls (patients: 8–22% vs. HCG: 5–14%, respectively). 17 patients and one healthy control did not receive a third vaccination (Table 2).

The adverse events most commonly described in both groups were pain around the injection site followed by fatigue, headaches, and muscle pain (Table 1). Only 2% [4] of patients showed an activation of their IRD. No severe adverse events were reported in either group.

**Fig. 1:**
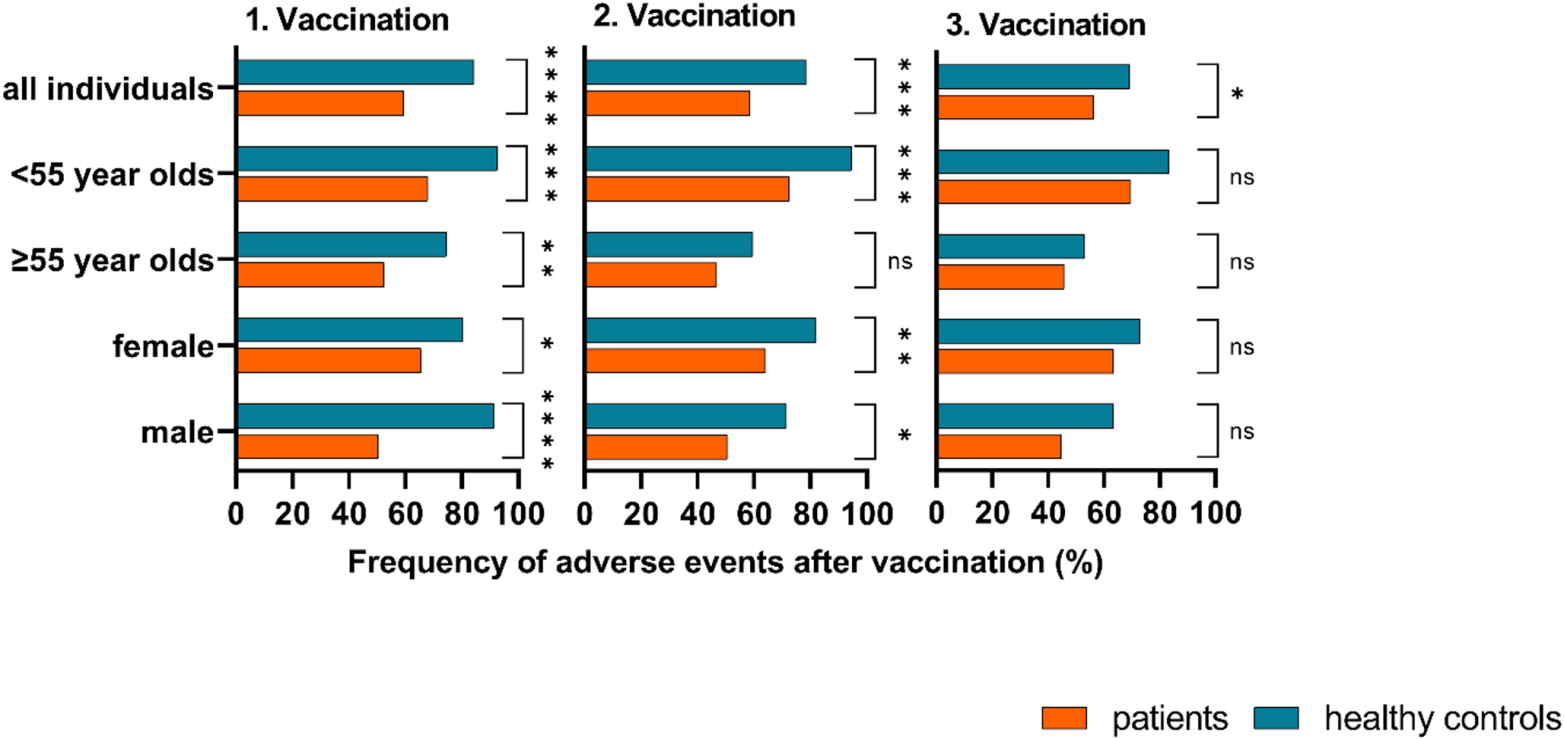
Percentage of patients and healthy controls experiencing adverse events after each vaccination against SARS-CoV-2 ns = not significant; *p < 0.05; **p < 0.01; ***p < 0.001; ****p < 0.0001

When comparing the relative amount of people experiencing adverse events after SARS-CoV-2 vaccination, significant differences between patients and healthy controls were found (Fig. 1). After the first vaccination, 59.6% (140) of the patients experienced any kind of adverse event, which is significantly lower compared to the 84.3% (86) seen in healthy controls (OR = 0.274 (95% CI: 0.151–0.497); *P* < 0.0001). After the second vaccination, the results were similar with 58.7% (138) of patients and 78.4% (80) of controls experiencing any adverse event (OR = 0.391 (0.228–0.670); *P* < 0.001). With 56.4% (123), the number of patients experiencing adverse events did not change after the third vaccination, but only 69.3% (70) of the healthy controls developed adverse events after their third vaccination. However, this still resulted in a significant difference between the two groups (OR = 0.573 (0.348–0.946); *P* = 0.029).

Dividing the study subjects in people < 55, and ≥ 55 years of age revealed significant differences between the age groups in either population. The < 55-year-old individuals were significantly more likely to experience adverse events after the first, second, and third vaccination compared to the ≥ 55-year-olds in both patients and controls (1. Vaccination: patients *P* = 0.016, HCG *P* = 0.011; 2. Vaccination: patients *P* < 0.0001, HCG *P* < 0.0001; 3. Vaccination: patients *P* < 0.001, HCG *P* = 0.002). However, differences between patients and healthy controls were still noticeable: Comparing the < 55-year-olds of both groups, 67.9% of patients, and 92.7% of the controls experienced adverse events after the first vaccination (OR = 0.166 (0.056–0.495); *P* < 0.001), 72.5% of the patients and 94.5% of the HCG experienced adverse events after the second vaccination (OR = 0.152 (0.044–0.524); *P* < 0.001), and 69.4% of patients and 83.3% of healthy controls developed adverse events after the third vaccination (*P* = 0.06). In individuals ≥ 55 years of age, the proportion of study subjects with adverse events was continuously lower throughout all vaccinations in the patients compared to the control group, however, this was only significant after the first vaccination (patients 52.4%; HCG 74.5%; OR = 0.377 (0.179–0.793); *P* = 0.009).

The difference between sexes was not as coherent as the differences in age. While female patients experienced adverse events significantly more often than the male patients after each vaccination (1. Vaccination: *P* = 0.02; 2. Vaccination: *P* = 0.04; 3. Vaccination: *P* = 0.006), this was not observed in the HCG. In fact, the proportion of females who experienced adverse events in the control group was not notably higher than that in male controls after any vaccination. However, female patients experienced adverse events significantly less frequently when compared to female controls after the first (65.5% and 80.6%, respectively; OR = 0.457 (0.227–0.918); *P* = 0.026) and second (64.1% and 82.1%, respectively; OR = 0.389 (0.191–0.794); *P* = 0.008) vaccination, while no notable difference was detected after the third vaccination. Similarly, in males 50.5% of the patients and 91.4% of the controls experienced adverse events after the first vaccination (OR = 0.096 (0.027–0.335); *P* < 0.0001), and 50.5% compared to 71.4% after the second vaccination (OR = 0.409 (0.177–0.945); *P* = 0.034). After the third vaccination the percentage of male controls experiencing adverse events was still notably higher than in the male patients, but significance was not reached.

**Fig. 2:**
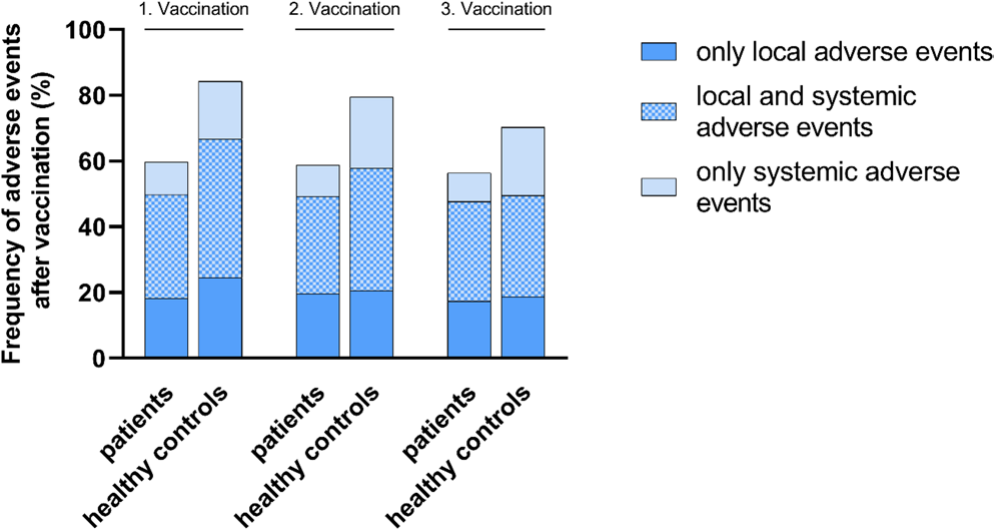
Frequency of adverse events after vaccination against SARS-CoV-2 in patients and healthy controls differentiated into “only local”, “local and systemic” and ”only systemic” events. The proportion of patients experiencing systemic events compared to the controls was significantly different after first, second, and third vaccination (*P* = 0.002, *P* < 0.001, *P* = 0.036). The number of healthy controls experiencing adverse events after the third vaccination was significantly lower compared to the second vaccination (*p* = 0.029)

Adverse events after vaccination can be differentiated into two subgroups, local and systemic adverse events (Fig. 2). The number of subjects who only experienced local adverse events did not seem to differ between patients and controls. However, patients seemed less likely to experience systemic events compared to the controls after all three vaccinations. 97 (41.3%) and 61 (59.8%) in patients and controls, respectively, after the first vaccination (OR = 0.472 [0.294–0.759]; *P* = 0.002), 91 (38.7%) compared to 60 (58.8%) after the second vaccination (OR = 0.442 [0.275–0.710]; *P* < 0.001), and 85 (39.0%) compared to 52 (51.5%) after third vaccination (OR = 0.602 [0.374–0.969]; *P* = 0.036).

While the frequency of adverse events did not change depending on the number of vaccinations in patients, there was a significant reduction of control subjects experiencing adverse events after the third compared to the second vaccination (*P* = 0.029) (Fig. 2).

The relative number of patients who experienced adverse events after vaccination was continuously 10–20% higher in patients who received mRNA-1273 compared to other vaccines but this difference was not significant probably due to the small sample size.

Finally, there was no association of the nature of the underlying IRD, its treatment or the application route (e.g. oral or subcutaneous) with reported adverse events.

## Discussion

Vaccination is the most appropriate and effective means of preventing severe consequences of SARS-CoV-2 infection. However, from the onset of global vaccination, there have been concerns about whether certain individuals, such as those with conditions requiring immunomodulatory and/or immunosuppressive therapies, would be particularly at risk from the vaccination itself. This was not least because experience with the novel principle of vaccination, i.e. mRNA-based vaccination, was lacking. Our aim was to gain a better understanding of the effects and side effects of SARS-CoV-2 vaccination in patients with IRDs and possibly to highlight differences in this regard compared with healthy individuals.

There already are studies that have been conducted on adverse events following SARS-CoV-2 vaccination, including one with over 10,000 participants from 94 countries [6, 7]. This study presents only minor differences between patients with IRDs and controls, supporting the recommendation of vaccination for these patients. Such an extensive study is valuable in demonstrating the safety of the vaccines and providing data on rare adverse events. However, different social structures or the different medical care before and after vaccination that come with such a heterogenic study group might dilute certain differences that would otherwise be apparent. This is where a rather homogeneous, single-centre study like ours can be useful in providing additional insight.

Similar to the previous studies, we did not find patients with IRDs to be at high risk of severe adverse events and could show that the vaccines were safe to use in patients. However, in our population it was noticeable that the proportion of individuals who experienced only local effects at the injection site was not different in the two study groups. In contrast, the probability of the occurrence of systemic effects was significantly lower in patients compared to controls. The systemic effects that occurred after vaccination, such as fever, muscle and joint pain, fatigue, or tiredness, may resemble the systemic symptoms that occur in active IRDs. Therefore, patients may not have associated these effects with vaccination or may not have registered them as unusual, unlike the healthy population, which is not accustomed to such symptoms. Another likely explanation could be the patients’ ongoing treatment. With glucocorticoids, methotrexate, or other immunomodulatory drugs, IRD patients receive comprehensive therapy designed to prevent clinical symptoms of systemic inflammation. It is reasonable to assume that this has influenced the frequency of such symptoms after vaccination.

To properly evaluate the results of our study, it is important to consider whether our cohorts had a different composition with individuals shown to be at higher risk for the occurrence of adverse events. For example, studies in healthy individuals have shown that younger age and female sex are risk factors for a more frequent occurrence of adverse events [8]. Consistent with the literature adverse events in our cohort were reported more commonly in individuals younger than 55 years of age and in female subjects compared to their male and older counterparts, respectively. Nonetheless, significant differences between patients and controls remained evident when grouped based on age or sex.

Another risk factor for the occurrence of adverse events is the vaccine used. mRNA-1273 causes systemic adverse events significantly more often than BNT162b2 [9, 10]. A similar observation was made in our study population. However, this does not affect our results negatively as the patient group had a higher proportion of individuals who received mRNA-1273 for each vaccination compared to the healthy controls.

While the scope of adverse events occurring after vaccination has by now been extensively shown, previous studies have not yet examined the changes in occurrence of adverse events during repeated vaccination in patients. Interestingly, the differences between the relative number of patients and controls who experienced systemic adverse events decreased with each subsequent vaccination. While the probability of adverse events in patients remained constant during repeated vaccinations, the proportion of healthy individuals experiencing adverse events decreased significantly after the third vaccination compared to the second vaccination. This is consistent with a CDC study of over 700,000 individuals who were less likely to report systemic reactions after a homologous booster vaccination than after the previous vaccination [11]. One explanation for the decrease in systemic adverse events after the third vaccination could be that successful vaccination had induced immunological memory that should neutralize the antigen quickly and without a vigorous systemic immune response upon re-exposure. However, further studies are needed to understand this change in the frequency of adverse events. It should be noted that this observation is not a statement against the benefit of booster vaccinations, as many previous studies have shown that booster vaccinations increase the immune response against SARS-CoV-2 and further boost immunity [12–14]. The fact that a reduction in systemic side effects was not observed in patients after the third vaccination may be due to the fact that they already had a lower frequency of systemic reactions after the first two vaccinations.

A possible bias in the results of our study could be that healthcare workers may have a special, professionally trained awareness of the development of side effects and therefore may have reported the occurrence of symptoms more frequently. We cannot completely rule out this possibility. However, since vaccination was safe in patients with IRDs undergoing immunosuppressive therapy in our cohort, we consider the results compiled here to be important in light of rising public concerns about the risks of vaccination against SARS-CoV-2, even if the difference in the occurrence of side effects compared to an unprimed healthy population may not be quite as clear in favor of the patients.

While differences between patients and controls were found in our study, the sample size of the study was still limited. Analysis of further subgroups was not possible and should be the focus of future studies. Furthermore, a reporting bias cannot be excluded since the participants reported adverse events subjectively. Since the control group mostly consisted out of healthcare workers, they might have been more aware of adverse events than patients. The study population was predominantly of white ethnicity limiting a transfer of the results to the general population. The findings presented in this study solely focused on adverse events and not the efficacy of the SARS-CoV-2 vaccinations in patients with IRDs. Analysis of the vaccine efficacy in a subgroup of the study population is still ongoing.

The results of our study show that repeated vaccinations against SARS-CoV-2 are safe in patients with inflammatory rheumatic diseases and that fears among patients and their doctors regarding specific side effects are unfounded. This is important in light of the ongoing pandemic and the continuing, and indeed increasing, reluctance to revaccinate for protection against the constantly mutating virus, especially among patients with inflammatory rheumatic diseases [15–17].

## Conclusion

Vaccination against SARS-CoV-2 was very well tolerated in patients with IRDs. Patients showed a lower risk for systemic adverse events than healthy controls. Importantly, serious events or activation of IRDs rarely occurred. As in healthy individuals, younger and female patients were more likely to report adverse events. The knowledge of the risks and specific risk factors should allow comprehensive and confident counselling of patients with IRDs on vaccination against SARS-CoV-2.

## Supplementary Information

Below is the link to the electronic supplementary material.

Supplementary Material 1

Supplementary Material 2

## Data Availability

For anonymity-reasons the data sets cannot be made publicly available. Confidential access might be granted upon reasonable request. Applications for accessing the data should be sent to the authors.
